# Real-world data analysis of perioperative chemotherapy patterns, G-CSF use, and FN status in patients with early breast cancer

**DOI:** 10.1007/s10549-023-07015-w

**Published:** 2023-07-06

**Authors:** Nobuhiro Shibata, Tetsuhiro Yoshinami, Kentaro Tamaki, Tomoyuki Nukada, Shinji Ohno

**Affiliations:** 1 Cancer Treatment Center, Kansai Medical University Hospital, 2-3-1, Shinmachi, Hirakata City, Osaka 573-1191 Japan; 2 Department of Breast and Endocrine Surgery, Osaka University Graduate School of Medicine, Suita, Osaka Japan; 3 Department of Breast Surgery, Nahanishi Clinic, Okinawa, Japan; 4 Kyowa Kirin Co., Ltd, Tokyo, Japan; 5 Breast Oncology Center, The Cancer Institute Hospital of the Japanese Foundation for Cancer Research, Tokyo, Japan

**Keywords:** Breast cancer, Perioperative chemotherapy, Febrile neutropenia, G-CSF, Pegfilgrastim, Real-world data

## Abstract

**Purpose:**

This study aimed to describe perioperative chemotherapy patterns, granulocyte colony-stimulating factor (G-CSF) use, and febrile neutropenia (FN) status in patients with early breast cancer (EBC) using real-world data in Japan.

**Methods:**

This retrospective observational study used anonymized claims data. The included patients were ≥ 18 years old, were female, and had breast cancer diagnosis and surgery records between January 2010 and April 2020. Measures included perioperative chemotherapy, G-CSF use (daily and primary prophylaxis [PP]), and FN and FN-related hospitalization (FNH), all examined annually. Perioperative chemotherapy was examined separately for human epidermal growth factor receptor 2-positive/negative (HER2±). A multivariate logistic regression was used to explore the factors associated with FNH.

**Results:**

Of 32,597 patients, those with HER2 + EBC treated with anthracycline-based regimens followed by taxane + trastuzumab + pertuzumab increased since 2018, and those with HER2 − EBC treated with doxorubicin/epirubicin + cyclophosphamide followed by taxane and dose-dense regimens increased after 2014. The proportion of patients prescribed daily G-CSF declined after 2014, whereas that of pegfilgrastim PP increased. The incidence proportion of FN remained at approximately 24–31% from 2010 to 2020, while that of FNH declined from 14.5 to 4.0%. The odds of FNH were higher in those aged ≥ 65 years and lower with pegfilgrastim PP administration.

**Conclusion:**

Despite the increasing use of escalated regimens in the last 5–6 years, FNH continuously declined, and the odds of FNH were lower among patients treated with pegfilgrastim PP. These results may suggest the contribution of PP in part to suppressing FNH levels over the last 5–6 years.

**Supplementary Information:**

The online version contains supplementary material available at 10.1007/s10549-023-07015-w.

## Introduction

Perioperative chemotherapy is one of the effective adjuvant systemic therapies for reducing the recurrence risk and mortality rates in patients with early breast cancer (EBC) [1]. The treatment landscape of chemotherapy for EBC has changed substantially in the last few decades, and adjuvant systemic therapy is currently tailored to breast cancer (BC) subtypes [2, 3]. Additionally, since the 2017 St. Gallen International Expert Consensus Conference [4], de-escalation and escalation of treatments [5] including dose-dense (dd) regimens [6] have been recommended when determined to be optimal. These changes have expanded treatment options for EBC.

Febrile neutropenia (FN) is a serious adverse event induced by myelosuppressive chemotherapy, and, in some cases, requires immediate hospitalization. FN can lead to dose reduction and treatment delays, which may compromise treatment outcomes [7]. Granulocyte colony-stimulating factors (G-CSFs) including pegfilgrastim, filgrastim, and lenograstim have been shown to reduce FN incidence [8]. Currently, four G-CSFs are available in Japan: filgrastim, lenograstim, and nartograstim, all of that require daily injections, and a long-lasting G-CSF, pegfilgrastim, that requires a single dose per cycle of chemotherapy [9]. With the changing treatment landscape for EBC, little is known regarding the patterns of real-world pegfilgrastim use after the introduction of pegfilgrastim in 2014 in Japan. Additionally, although FN risk according to some regimens has recently been reported [10], comprehensive nationwide reports including the years before the introduction of pegfilgrastim are not available.

This real-world data analysis aimed to describe perioperative chemotherapy patterns, G-CSF use, and FN status from 2010 to 2020 in patients with EBC in clinical practice in Japan using a nationwide claims database. Additionally, we explored the factors associated with FN-related hospitalization (FNH) using a multivariate logistic regression model.

## Methods

### Study design and data source

This retrospective observational study used anonymized, secondary data originally recorded for insurance reimbursement purposes. Data from 1 January 2010 to 31 October 2020 were extracted from a medical claims database managed by Medical Data Vision Co., Ltd. (MDV, Tokyo, Japan). The database covers approximately 24% of nationwide medical institutions with emergency departments participating in the diagnosis procedure combination/per-diem payment system (DPC/PDPS), storing individual-level data from approximately 34.51 million patients at 436 medical institutions (as of October 2020). The data include demographics (e.g., age and sex), diagnosis codes (International Statistical Classification of Diseases and Related Health Problems, 10th Revision [ICD-10]), claims, and laboratory data (for some institutions) of both inpatients and outpatients.

The study protocol was approved by the Research Institute of Healthcare Data Science (RI2021015). According to the “Ethical Guidelines for Medical and Health Research Involving Human Subjects”, a study using secondary data stored in an anonymized structured format, such as this study, is outside the scope of the guidelines. Informed consent was not required for this study. This study was registered with the University Hospital Medical Information Network Clinical Trial Registry (UMIN000046199).

### Patient selection

Included patients were those with records of BC diagnosis (ICD-10: C50.) and surgery in the month of the first BC diagnosis or later identified between 1 January 2010 and 30 April 2020. The end month (April 2020) was selected to extract sufficient data considering a typical 6-month chemotherapy regimen. The excluded patients were < 18 years old, male, with records of cancer diagnosis other than BC and chemotherapy or radiotherapy within 1 year before the first BC diagnosis, and without chemotherapy records.

### Measures

Measures included chemotherapy, and combination regimens were defined as drugs administered within 3 days before and after administration of one drug (for 7 days in total; drugs described below), and each regimen was considered to end when no chemotherapy record was identified for 30 days from the end of the previous cycle. A regimen was defined as dd when at least two cycles were performed at ≤ 20-day-intervals. The duration and number of cycles for the regimens are provided in Supplementary Table S1. Up to the first two chemotherapy regimens were identified for each patient from the first BC diagnosis to the month of surgery, or during the 6 months following the month of surgery. The two separate regimens were presented with a hyphen (“−”). Chemotherapy regimens included anthracycline—taxane + trastuzumab (A-Taxane + H), anthracycline—taxane + trastuzumab + pertuzumab (A-Taxane + HP), docetaxel + trastuzumab (DH), docetaxel + cyclophosphamide + trastuzumab (TCH), docetaxel + carboplatin + trastuzumab (TCbH), docetaxel + carboplatin + trastuzumab + pertuzumab (TCbHP), paclitaxel + trastuzumab (TH), taxane + trastuzumab + pertuzumab (Taxane + HP), doxorubicin/epirubicin + cyclophosphamide (AC/EC), cyclophosphamide (oral) + methotrexate + fluorouracil (CMF), fluorouracil + epirubicin + cyclophosphamide (FEC), docetaxel + doxorubicin + cyclophosphamide (TAC), docetaxel + cyclophosphamide (TC), dose-dense (dd), and other. The “anthracycline” included AC/EC, dd AC/EC, FEC, and cyclophosphamide (oral) + epirubicin + fluorouracil. The “taxane” included docetaxel (DTX), TC, paclitaxel (PTX: weekly, every 2 weeks, and every 3 weeks), and nab-PTX (every 3 weeks).

Chemotherapy was defined as complete if the number of cycles performed was equal to or greater than the number specified in Supplementary Table S1; otherwise the regimen was defined as discontinued. Completed chemotherapy was further assessed with and without delay; a regimen was defined as delayed if any subsequent cycle after the first cycle started ≥ 7 days after the scheduled cycle initiation. In cases where two regimens were identified, both regimens meeting either of the above definitions were defined as completed or delayed. The timing of chemotherapy (preoperative and postoperative) was also assessed.

Daily G-CSF included filgrastim, lenograstim, or nartograstim administered on day 6 or later of each cycle initiation (day 1). Pegfilgrastim was administered as the primary and secondary prophylaxis (PP and SP, respectively). PP was defined as the drug administered on days 1–5 of the first cycle initiation (day 1). SP was defined as pegfilgrastim not administered during the first cycle and administered on days 1–5 of the second or later cycle initiation (day 1).

FN was defined using ICD-10 codes (D70) and disease codes (8842350) identified during perioperative chemotherapy. FNH was identified using any of the following codes on hospital admissions: 1) FN; 2) sepsis (ICD-10: A02, A32, A39-41, B37, I30, I33, J02, J20, L02, L08, M86, O85, T81) and intravenous antibacterial drugs (ATC codes: J01A-H, J01K, J01P, and J01X); 3) infections (ICD-10: A01-09, A15-19, A23, A25-28, A30-32, A35-43, A46, A48, A49, B35-38, B42-49, and B99) and intravenous antibacterial drugs; and 4) fever (ICD-10: R50) and intravenous antibacterial drugs.

### Statistical analysis

Patient background characteristics were descriptively analyzed. The chemotherapy regimens were examined annually from 2010 to 2020. If a series of regimen cycles encompassed 2 years, it was counted in the earlier year when the first cycle was initiated. Regimens were stratified by human epidermal growth factor receptor 2-positive/negative (HER2±), and the latter was further stratified by estrogen receptor-positive/negative (ER±). HER2 + and HER2− were defined depending on the presence or absence of anti-HER2 therapy records in the month of the first BC diagnosis or later, respectively. ER+ and ER− were defined depending on the presence or absence of selective estrogen receptor modulator (tamoxifen and toremifene) or aromatase inhibitor (anastrozole, exemestane, and letrozole) records in the month of the first BC diagnosis or later, respectively. Chemotherapy completion/discontinuation and with/without delay in patients who completed chemotherapy were assessed for the overall population. The timing of chemotherapy (preoperative and postoperative) was examined annually with stratification by the BC subtype. G-CSF use (daily, PP, and SP) was examined annually in the overall population and was additionally stratified by regimen. We examined regimens used by ≥ 1,000 patients or regimens whose percentage of patients treated with pegfilgrastim PP exceeded 50% in any year. The incidence proportion of FN and FNH was examined annually in the overall population and was additionally stratified by regimen separately for patients with HER2+ and HER2−.

Factors associated with FNH were examined using a logistic regression model, and the explanatory variables were regimens (AC/EC, FEC, DTX + HP, TCH, TC, DTX, TH, dd AC/EC, TCbH, and DH), age (< 65 and ≥ 65 years), time from BC surgery to the start of chemotherapy (before surgery, ≤ 30 days, 31–60 days, and ≥ 61 days), comorbidity (cardiovascular disease, renal disease, liver disease, diabetes, and human immunodeficiency virus or acquired immunodeficiency syndrome) identified within 1 year before BC diagnosis (0, 1, and ≥ 2), and pegfilgrastim PP (no administration and administration). When two regimens were identified in a patient, FNH/no FNH during the first regimen was assessed. In this exploratory analysis, we used the outcome FNH defined based on the combination records because we speculate that FN codes would include various FN severities. The FN codes were used to gain broad understanding of treatment patterns for FN. This analysis focused on the first FN involving hospital admission. Additionally, a post-hoc analysis was conducted to examine the impact of postoperative radiation therapy on FNH. We have performed this logistic regression model using the same explanatory variables as used in the main analysis (described above) plus the postoperative radiation therapy (not performed and performed [performed ≥ 5 times from the date of surgery to the date before the initiation of the first chemotherapy or radiotherapy performed ≥ 5 times during the first chemotherapy after surgery]).

Missing data were not imputed, and all statistical analyses were performed using SAS Release 9.4 or later (SAS Institute, NC, USA).

## Results

### Study population

**Fig. 1:**
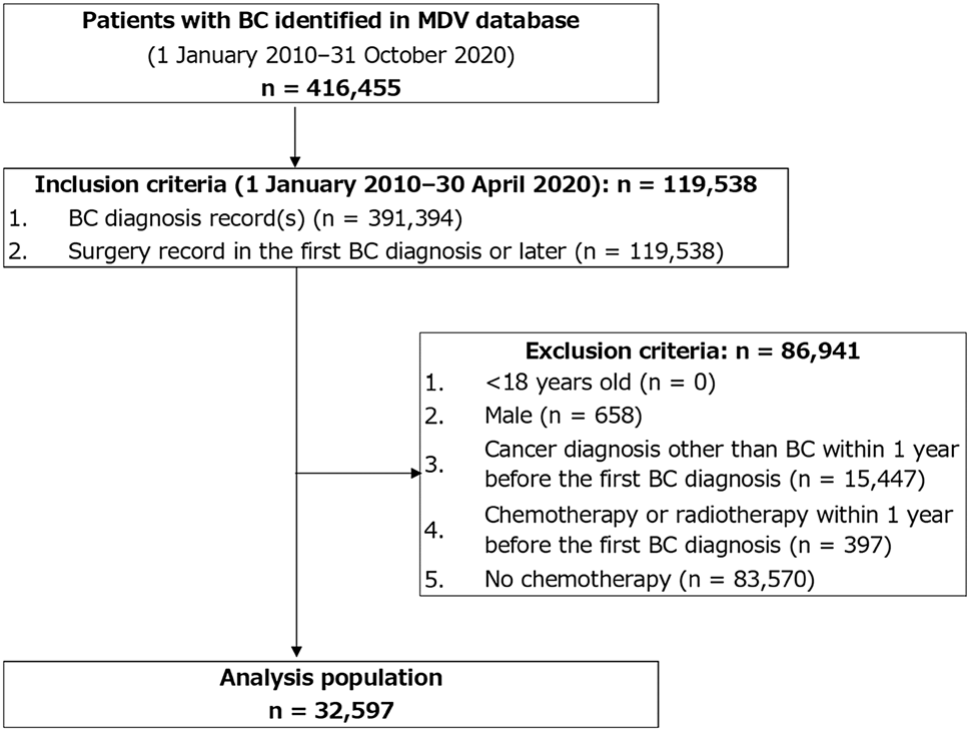
Patient flow diagram. *BC* breast cancer, *MDV* Medical Data Vision Co., Ltd

BC diagnosis records were identified in 416,455 patients in the MDV database (Fig. 1). Of these patients, 119,538 satisfied the inclusion criteria, and 86,941 were excluded, most of whom were due to without chemotherapy (n = 83,570) followed by, although not mutually exclusive, other cancer diagnosis within 1 year before the first BC diagnosis (n = 15,447), male (n = 658), and chemotherapy or radiotherapy provided within 1 year before the first BC diagnosis (n = 397). The analysis population comprised 32,597 patients. The mean ± SD age of the overall population was 55.7 ± 11.5 years, and most patients were < 65 years old (74.0%) (Table 1). The most common BC subtype was ER+/HER2− (44.2%), followed by ER−/HER2− (22.5%), ER+/HER2+ (18.7%), and ER−/HER2+ (14.7%). The majority of the patients did not have any comorbidities examined (98.9%).

**Table 1:** Patient characteristics

Variables	Category	Overall	
		n = 32,597	
		n	(%)
Age, year	Mean ± SD	55.7 ± 11.5	
	Median	56	
	Minimum, maximum	19, 90	
	< 65	24,112	(74.0)
	≥ 65	8485	(26.0)
Subtype ^a,b^	ER+/HER2−	14,392	(44.2)
	ER+/HER2+	6099	(18.7)
	ER−/HER2+	4784	(14.7)
	ER−/HER2−	7,322	(22.5)
Cardiovascular disease	No	32,547	(99.8)
	Yes	50	(0.2)
Renal disease	No	32,590	(100.0)
	Yes	7	(0.0)
Liver disease	No	32,419	(99.5)
	Yes	178	(0.5)
Diabetes	No	32,410	(99.4)
	Yes	187	(0.6)
HIV or AIDS	No	32,597	(100.0)
	Yes	0	(0.0)
Number of comorbidity ^c^ within 1 year before BC diagnosis ^b^	0	32,225	(98.9)
	1	323	(1.0)
	≥ 2	49	(0.2)
Timing of chemotherapy	Before surgery	12,156	(37.3)
	After surgery	20,441	(62.7)

*SD* standard deviation, *ER* estrogen receptor, *HER2* human epidermal growth factor receptor 2, *HIV* human immunodeficiency virus, *AIDS* acquired immunodeficiency syndrome, *BC* breast cancer

^a^The BC subtype was defined depending on the presence or absence of selective estrogen receptor modulators (tamoxifen and toremifene) or aromatase inhibitors (anastrozole, exemestane, and letrozole) in the month of the first BC diagnosis or later (ER+ and ER − , respectively) and anti-HER2 therapies in the month of the first BC diagnosis or later (HER2+ and HER2−, respectively)

^b^The percentages do not add up to 100% due to rounding

^c^The comorbidity included cardiovascular disease, renal disease, liver disease, diabetes, and HIV or AIDS

### Chemotherapy

**Fig. 2:**
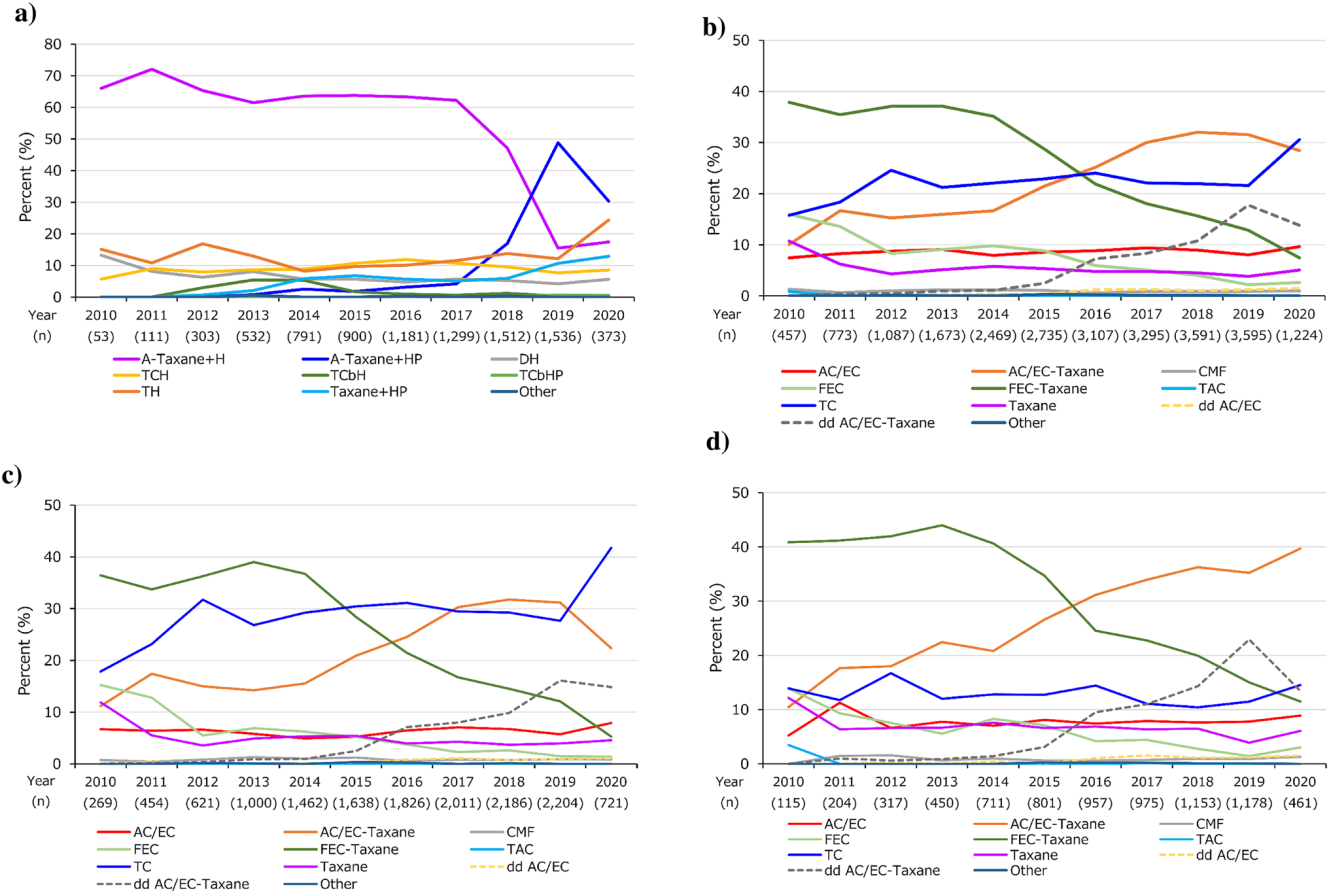
Annual perioperative chemotherapy regimens stratified by breast cancer subtype: **a** HER2+, **b** HER2−, **c** ER+/HER2−, and **d** ER−/HER2−. *HER2* human epidermal growth factor receptor 2, *ER* estrogen receptor. Notes: The figure shows up to the first two perioperative regimens, and two regimens, if present, were presented with a hyphen (“-”). Regimens were as follows; A: anthracycline; Taxane: docetaxel, docetaxel + cyclophosphamide (TC), paclitaxel (PTX: weekly, every 2 weeks, and every 3 weeks), and nab-PTX (every 3 weeks); H: trastuzumab; HP: trastuzumab + pertuzumab; DH: docetaxel + trastuzumab; TCH: docetaxel + cyclophosphamide + trastuzumab; TCbH: docetaxel + carboplatin + trastuzumab; TCbHP: docetaxel + carboplatin + trastuzumab + pertuzumab; TH: paclitaxel + trastuzumab; AC/EC: doxorubicin/epirubicin + cyclophosphamide; CMF: cyclophosphamide (oral) + methotrexate + fluorouracil; FEC: fluorouracil + epirubicin + cyclophosphamide; TAC: docetaxel + doxorubicin + cyclophosphamide; and dd: dose-dense. The anthracycline included AC/EC, dd AC/EC, FEC, and cyclophosphamide (oral) + epirubicin + fluorouracil. The data in 2020 included patients who had BC diagnosis records and surgery records in the month of the first BC diagnosis or later identified until 30 April 2020

The proportion of HER2 + patients treated with A-Taxane + H was over 60% from 2010 to 2017, and the number dropped thereafter (66.0% in 2010 to 17.4% in 2020) (Fig. 2a). Conversely, the proportion of those treated with A-Taxane + HP was low until 2017 and increased thereafter (30.3% in 2020). The TCH, TH, and Taxane + HP regimens were used by up to approximately 24% of the patients. Regimens including carboplatin (TCbH and TCbHP) were rarely used during the 11 years.

For HER2− patients, the proportion of those treated with FEC-Taxane and FEC decreased from 2010 to 2020 (37.9% to 7.4% and 16.0% to 2.6%, respectively) (Fig. 2b). The proportion of patients treated with AC/EC was low but remained relatively stable over the 11 years, whereas the proportion of those treated with AC/EC-Taxane and TC increased from 2010 to 2020 (10.1% to 28.4% and 15.8% to 30.6%, respectively). The dd regimens were rarely used until 2014, but dd AC/EC-Taxane use gradually increased thereafter, with a slight decline in 2020.

Similar to the overall results for HER2−, the proportion of ER+/HER2− patients treated with FEC-Taxane and FEC declined from 2010 to 2020, whereas the proportion of those treated with AC/EC-Taxane and TC increased (Fig. 2c). A similar increasing pattern in dd AC/EC-Taxane as for the overall HER2− was observed. For ER−/HER2− patients, the trends were generally similar to those of the overall HER2− (Fig. 2d). The FEC-Taxane was administered to over 40% of the patients by 2014, and subsequently decreased to 11.5% in 2020. The proportion of patients treated with FEC also decreased over the 11 years (13.9% to 3.0%). The AC/EC-Taxane use increased, while TC use remained stable over the 11 years. A similar increasing pattern in dd AC/EC-Taxane as for the overall HER2− was observed.

Regarding chemotherapy completion/discontinuation, less than 30% of patients discontinued most regimens, and a relatively small proportion of patients who completed chemotherapy delayed chemotherapy (Supplementary Figure S1). Regarding the timing of chemotherapy, the proportion of HER2 + patients treated with preoperative chemotherapy was slightly higher than that of HER2− patients, and the proportion was the highest in ER−/HER2+ patients, followed by ER−/HER2− patients (Supplementary Figure S2).

### G-CSF

Of the overall population, 23.1% were prescribed daily G-CSF in 2010, and this level was relatively stable at approximately 23–30% until 2014 and gradually declined thereafter (16.6% in 2020, Fig. 3). Conversely, the proportion of patients administered pegfilgrastim as PP increased from 2014 to 2020 (0.8% to 44.8%). G-CSF use stratified by chemotherapy regimens showed similarly increasing patterns of pegfilgrastim PP administration in most subgroups after 2014 (Supplementary Figure S3). Approximately 40%–60% of HER2+ patients treated with TCH were administered pegfilgrastim as PP in the last 5 years (Supplementary Figure S3a). For the A-Taxane + H and A-Taxane + HP regimens, approximately 25%–50% of the patients were treated with PP. Among HER2− patients, approximately 50%–90% treated with dd regimens were administered pegfilgrastim as PP in the last 5 years (Supplementary Figure S3b). Approximately 35%–60% of the patients treated with TC and 20–40% of the patients treated with AC/EC or FEC both ± Taxane were administered PP, respectively.

**Fig. 3:**
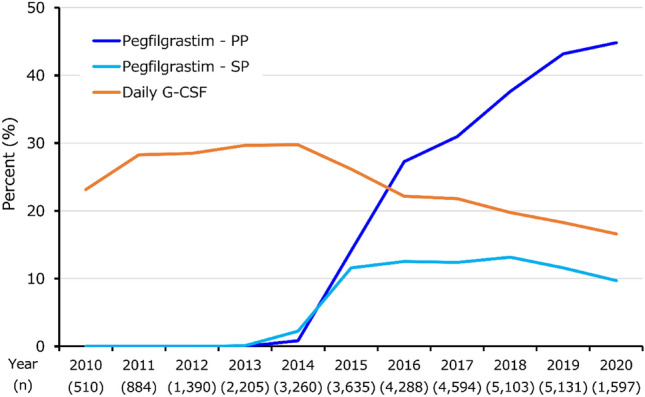
Annual G-CSF use. *G-CSF* granulocyte colony-stimulating factor, *PP* primary prophylaxis, *SP* secondary prophylaxis

### FN and FNH

**Fig. 4:**
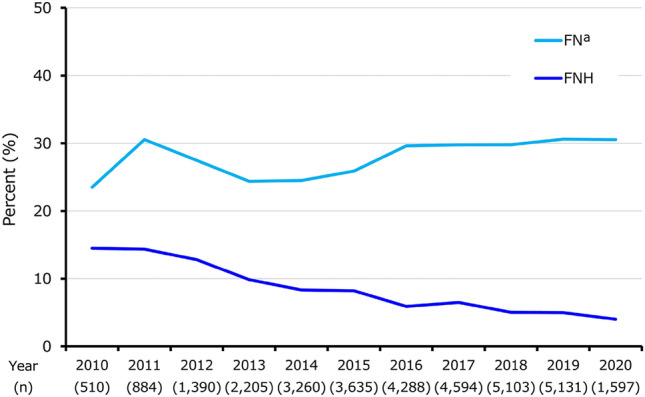
Annual FN and FNH. *FN* febrile neutropenia, *FNH* febrile neutropenia-related hospitalization, *ICD-10* International Statistical Classification of Diseases and Related Health Problems, 10th Revision. Notes: ^a^FN was defined based on disease codes (ICD-10: D70 and disease code: 8842350)

In the overall population, the incidence proportion of FN remained stable at approximately 24%–31% over the 11 years, while the proportion of those with FNH declined from 14.5% in 2010 to 4.0% in 2020 (Fig. 4). With respect to the stratification by regimen among patients with HER2 + , the incidence proportion of FN was relatively higher in DTX + HP and TCH for single regimens, and in dd regimens for the two regimens (Supplementary Table S2a). Among HER2− patients, the proportion of those with FN was relatively higher in dd AC/EC and TC for single regimens, and dd regimens for the two regimens (Supplementary Table S2b). However, the proportion of patients with FNH was not substantially higher in these regimens for both HER2+ and HER2−.

### Factors associated with FNH

The baseline characteristics of the patients included in the logistic regression model and the ORs for FNH are shown in Table 2. The odds of FNH were higher in the following categories: regimens of FEC (OR [95% CI] 1.878 [1.679–2.100]), DTX + HP (1.687 [1.187–2.399]), TCH (1.385 [1.050–1.827]), and TC (1.281 [1.117–1.470]); and age ≥ 65 years (1.268 [1.147–1.403]). The odds of FNH were lower in the following categories: regimens of DTX (0.430 [0.293–0.632]) and TH (0.235 [0.135–0.410]); ≤ 30 days from BC surgery to the start of chemotherapy (0.773 [0.657–0.910]); and pegfilgrastim administration as PP (0.879 [0.778–0.993]). The post-hoc logistic analysis showed generally similar results to those of the main analysis (Supplementary Table S3). Additionally, the odds of FNH were similar in patients not performed and performed post-operative radiation therapy.

**Table 2:** Baseline characteristics of patients included in the logistic regression model and the OR of FNH

Variables	Category	Overall	FNH		No FNH		OR	95% CI		*p*-value (Wald Chisq)
		n = 28,476	n = 2,102		n = 26,374					
		n	n	(%)	n	(%)		LCL	UCL	
Regimen ^a^	AC/EC	9,748	589	(6.0)	9,159	(94.0)	1.000	–	–	–
	FEC	7,574	812	(10.7)	6,762	(89.3)	1.878	1.679	2.100	< .0001
	DTX + HP	368	37	(10.1)	331	(89.9)	1.687	1.187	2.399	0.0014
	TCH	764	61	(8.0)	703	(92.0)	1.385	1.050	1.827	0.0126
	TC	5,215	390	(7.5)	4,825	(92.5)	1.281	1.117	1.470	0.0007
	DTX	1,022	28	(2.7)	994	(97.3)	0.430	0.293	0.632	< .0001
	TH	818	13	(1.6)	805	(98.4)	0.235	0.135	0.410	< .0001
	dd AC/EC	2,218	122	(5.5)	2,096	(94.5)	0.997	0.805	1.234	0.9911
	TCbH	125	13	(10.4)	112	(89.6)	1.764	0.987	3.153	0.0328
	DH	624	37	(5.9)	587	(94.1)	0.957	0.679	1.349	0.8064
Age, years	< 65	21,357	1,512	(7.1)	19,845	(92.9)	1.000	–	–	–
	≥ 65	7,119	590	(8.3)	6,529	(91.7)	1.268	1.147	1.403	< .0001
Time from breast cancer surgery to the start of chemotherapy, day	Before surgery	9,817	758	(7.7)	9,059	(92.3)	0.962	0.863	1.071	0.1392
	≤ 30	3,157	191	(6.1)	2,966	(93.9)	0.773	0.657	0.910	0.0086
	31–60	11,527	877	(7.6)	10,650	(92.4)	1.000	–	–	–
	≥ 61	3,975	276	(6.9)	3,699	(93.1)	0.902	0.784	1.039	0.9553
Comorbidity ^b^ within 1 year before breast cancer diagnosis	0	28,157	2,088	(7.4)	26,069	(92.6)	1.000	–	–	–
	1	280	13	(4.6)	267	(95.4)	0.619	0.353	1.084	0.8242
	≥ 2	39	1	(2.6)	38	(97.4)	0.295	0.040	2.161	0.3394
Pegfilgrastim PP	No administration	22,201	1,692	(7.6)	20,509	(92.4)	1.000	–	–	-
	Administration	6,275	410	(6.5)	5,865	(93.5)	0.879	0.778	0.993	0.0384

*OR* odds ratio, *FNH* febrile neutropenia-related hospitalization, *CI* confidence interval, *LCL* lower confidence limit, *UCL* upper confidence limit, *PP* primary prophylaxis

^a^The first chemotherapy regimen was examined in each patient if two regimens were identified, and regimens were as follows: AC/EC: doxorubicin/epirubicin + cyclophosphamide; FEC: fluorouracil + epirubicin + cyclophosphamide; DTX + HP: docetaxel + trastuzumab + pertuzumab; TCH: docetaxel + cyclophosphamide + trastuzumab; TC: docetaxel + cyclophosphamide; DTX: docetaxel; TH: paclitaxel + trastuzumab; dd: dose-dense; TCbH: docetaxel + carboplatin + trastuzumab; and DH: docetaxel + trastuzumab

^b^The comorbidity included cardiovascular disease, renal disease, liver disease, diabetes, and human immunodeficiency virus or acquired immunodeficiency syndrome

## Discussion

This real-world data analysis is one of the first to examine perioperative chemotherapy patterns, G-CSF use, and FN status among patients with EBC from 2010 to 2020 using a nationwide claims database in Japan. We found that the number of patients with HER2 + treated with A-Taxane + HP increased since around 2018, and those with HER2− were increasingly treated with escalated regimens such as AC/EC-Taxane and dd regimens since around 2014. Pegfilgrastim administration as PP has increased since the introduction of pegfilgrastim in 2014. The proportion of patients with FNH continuously declined over the 11 years. Our logistic regression analysis showed that the odds of FNH were higher in some regimens such as FEC and TC than in AC/EC, and in patients aged ≥ 65 years than in those aged < 65 years, and lower in patients treated with pegfilgrastim PP.

The increasing use of A-Taxane + HP among patients with HER2+ since 2018 corresponds to the additional indication provided to pertuzumab in October 2018 for the treatment of HER2 + EBC [11]. For HER2−, patients have been increasingly treated with dd regimens since 2014. PP, enabled by pegfilgrastim approval in 2014, presumably allowed treatments using escalated regimens. Furthermore, a steeper increase in the dd regimen was observed after 2018, and this increase may reflect recommendations of dd regimens by the Japanese guidelines in 2018 as adjuvant chemotherapy for patients with high recurrence risk [3].

Additionally, trends in regimens such as FEC and TC were in line with the evidence previously presented. The regimens FEC-Taxane and FEC use, although to a lesser degree, declined during the 11-year period irrespective of estrogen receptor expression among patients with HER2−. Its decline around 2014 may be partly attributed to reports from a phase III Gruppo Italiano Mammella 2 trial in which the addition of fluorouracil to epirubicin, cyclophosphamide, and paclitaxel was compared to a regimen without fluorouracil for node-positive BC. The study found no significant improvements in disease-free survival (DFS) and overall survival (OS) at 5 years between the two groups [12]. Recently, the phase III, NSABP B-36 trial for node-negative invasive BC further demonstrated that 6-cycle FEC-100 (5-fluorouracil + epirubicin + cyclophosphamide) did not improve DFS or OS, relative to 4-cycle AC, and FEC-100 was associated with greater toxicity [13]. Furthermore, our real-world data showed that the regimen TC use gradually increased since 2010 among patients with ER+/HER2−, and the result was also in line with evidence previously presented in the US Oncology Research Trial 9735; among overall patients with HER2− and HER2 + , significant improvements in DFS and OS at 7 years were found in TC over AC [14].

Our results indicated that in most regimens two-thirds of the patients completed chemotherapy according to schedule (Supplementary Figure S1). As no reports on completion or delay regarding various regimens have been reported in Japan, these data provide insights into real-world therapy status and could guide clinicians in selecting optimal regimens for patients. Regarding the timing of chemotherapy, slightly increasing trends in preoperative chemotherapy were observed in HER2+ and ER−/HER2− patients (Supplementary Figure S2), in line with the current clinical response-guided approaches in neoadjuvant settings [2, 3, 5]. We note here that in 2020 almost all patients with any BC subtypes were postoperatively treated with chemotherapy, presumably due to the COVID-19 pandemic in various countries, including Japan. At the time, surgeries were postponed by many medical institutions.

Our data showed that the administration of pegfilgrastim as PP has increased substantially since the launch of pegfilgrastim in 2014. Furthermore, regimen-specific G-CSF use indicated that G-CSF was administered according to the FN risk in clinical practice per the recommendation. Pegfilgrastim was more commonly administered as PP in regimens such as TCH and TC (approximately 35–60%) and dd regimens (approximately 50–90%) (Supplementary Figure S3). The guidelines in Japan and overseas recommend PP for regimens with more than 20% FN incidence [15, 16], and the NCCN guidelines list these regimens as high risk for FN [17]. Additionally, we found that approximately 20–40% of patients treated with AC/EC or FEC both ± Taxane were administered pegfilgrastim as PP, and the NCCN guidelines list these regimens as intermediate risk for FN [17]. In contrast to increased pegfilgrastim administration as PP, daily G-CSF prescription has declined since around 2014, but was still prescribed to 16.6% of patients in 2020. Although the guidelines for G-CSF use in Japan do not recommend the therapeutic use of daily G-CSF, a certain number of patients may be treated therapeutically at the clinicians’ discretion.

The proportion of FN patients remained stable from 2010 to 2020. We examined FN identified based on diagnosis codes (ICD-10: D70 and disease code: 8842350) to obtain a broad understanding of treatments related to FN regardless of its severity. The observed annual FN trends therefore broadly reflect treatments for FN, and we consider that various FN severities would have been included in this FN estimate. Due to this reason, the observed FN trends must be carefully interpreted. In contrast to the relatively stable FN, the proportion of patients with FNH continuously declined from 2010 to 4.0% in 2020, despite the increasing use of escalated regimens in the last 5–6 years. Our multivariate logistic regression model showed that the odds of FNH were lower in patients administered pegfilgrastim PP. These results may indicate that PP using pegfilgrastim may have partly contributed to the declining trends in FNH. The results of other factors including age (≥ 65 years) and some regimens (e.g., AC/EC, FEC, and TC) were as expected; older age, among others including comorbidities such as cardiovascular disease and renal and liver dysfunction, are risk factors for developing FN [15–17], and the NCCN guidelines describe regimens such as TC as high FN risk and anthracycline regimens as intermediate FN risk [17].

### Limitations

This study has several limitations in terms of generalizability, validity of diagnosis/subtypes, and statistical analysis. First, the MDV database comprises data mainly recorded at acute, advanced care medical institutions that adopt DPC [18], and those that do not adopt DPC are not included. As BC treatments are generally provided at relatively large medical institutions that are also equipped for acute care, we consider bias in patient characteristics is limited. Second, we defined FN based on the ICD-10 and disease codes recorded for insurance claims purposes. These codes have not been validated and may not perfectly reflect patients’ medical conditions. The BC subtype was not recorded in the database and was extrapolated from the records of endocrine and anti-HER2 therapies. Lastly, we used variables that are potentially associated with FNH, but factors known to be associated with FNH (e.g., chemotherapy quantity/strength, performance status, blood parameters, and cancer stage) could not be incorporated into the statistical model because such information was not recorded in the database. Our exploratory statistical analysis included all data including the years before pegfilgrastim was available. This is because our aim was to gain broad understanding of factors associated with FNH. Additionally, owing to the retrospective observational study design, further research with prospective designs evaluating risk factors of FNH would be of clinical value.

## Conclusion

This real-world data analysis in Japan found that even after escalated regimens have been increasingly used in the last 5–6 years since the introduction of pegfilgrastim in 2014, FNH continuously declined. The number of patients treated with pegfilgrastim PP increased over the years, and a logistic regression model showed lower odds of FNH among patients treated with pegfilgrastim PP. These results may suggest the contribution of PP in part to suppressing the level of FNH over the last 5–6 years.

## Supplementary Information

Below is the link to the electronic supplementary material.

Supplementary file1 (DOCX 875 kb)

## Data Availability

The data that support the findings of this study are proprietary to Medical Data Vision Co., Ltd. (MDV), and restrictions apply to the availability of these data, which were used under license for the current study, and thus are not publicly available. The data are available for purchase from the MDV. For inquiries regarding data purchases, please contact ebm_sales@mdv.co.jp.
