# Supplementary material for: Real-world data analysis of perioperative chemotherapy patterns, G-CSF use, and FN status in patients with early breast cancer
Source: Breast Cancer Res Treat. 2023 Jul 6;201(2):265–73. doi: 10.1007/s10549-023-07015-w (PMC10361858; doi:10.1007/s10549-023-07015-w)
Supplement: Supplementary file 1 — Supplementary file1 (DOCX 875 kb) [file 10549_2023_7015_MOESM1_ESM.docx]

# **Title: Real-world data analysis of perioperative chemotherapy patterns, G-CSF use, and FN status in patients with early breast cancer**

**Journal name:** Breast Cancer Research and Treatment

**Authors:**

Nobuhiro Shibata^1,*^, Tetsuhiro Yoshinami^2^, Kentaro Tamaki^3^, Tomoyuki Nukada^4^, and Shinji Ohno^5^

**Author affiliations:**

^1^ Cancer Treatment Center, Kansai Medical University Hospital, Hirakata, Osaka, Japan

^2^ Department of Breast and Endocrine Surgery, Osaka University Graduate School of Medicine, Suita, Osaka, Japan

^3^ Department of Breast Surgery, Nahanishi Clinic, Okinawa, Japan

^4^ Kyowa Kirin Co., Ltd., Tokyo, Japan

^5^ Breast Oncology Center, The Cancer Institute Hospital of the Japanese Foundation for Cancer Research, Tokyo, Japan

*** Corresponding author:**

Nobuhiro Shibata

Cancer Treatment Center, Kansai Medical University Hospital

2-3-1, Shinmachi, Hirakata city, Osaka 573-1191 Japan

TEL: +81-72-804-0101

E-mail: [shibanob.kmu@gmail.com](mailto:shibanob.kmu@gmail.com)

# **Supplementary materials**

## **Supplementary Table S1** Duration of each cycle and number of cycles for early breast cancer

| **Regimen** | **Regimen (description)** | **Duration of each cycle (day)** | **Number of cycles** |
| --- | --- | --- | --- |
| AC | doxorubicin + cyclophosphamide | 21 | 4 |
| EC | epirubicin + cyclophosphamide | 21 | 4 |
| dd AC | dose-dense doxorubicin + cyclophosphamide | 14 | 4 |
| dd EC | dose-dense epirubicin + cyclophosphamide | 14 | 4 |
| FEC | fluorouracil + epirubicin + cyclophosphamide | 21 | 4 |
| CEF | cyclophosphamide (oral) + epirubicin + fluorouracil | 28 | 6 |
| DTX | docetaxel | 21 | 4 |
| TC | docetaxel + cyclophosphamide | 21 | 4 |
| PTXq1w | paclitaxel weekly | 7 | 12 |
| PTXq2w | paclitaxel every 2 weeks | 14 | 4 |
| PTXq3w | paclitaxel every 3 weeks | 21 | 4 |
| nab-PTX | nab-paclitaxel every 3 weeks | 21 | 4 |
| DH | docetaxel + trastuzumab | 21 | 4 |
| TCH | docetaxel + cyclophosphamide + trastuzumab | 21 | 4 |
| TCbH | docetaxel + carboplatin + trastuzumab | 21 | 6 |
| TCbHP | docetaxel + carboplatin + trastuzumab + pertuzumab | 21 | 6 |
| TH | paclitaxel + trastuzumab | 21 | 4 |
| CMF | cyclophosphamide (oral) + methotrexate + fluorouracil | 28 | 6 |
| TAC | docetaxel + doxorubicin + cyclophosphamide | 21 | 4 |
| DTX+HP | docetaxel + trastuzumab + pertuzumab | 21 | 4 |
| THP | paclitaxel + trastuzumab + pertuzumab | 21 | 4 |
| CAF | cyclophosphamide (oral) + doxorubicin + fluorouracil | 28 | 6 |
| FAC | fluorouracil + doxorubicin + cyclophosphamide | 21 | 4 |

## **Supplementary Figure S1** Chemotherapy completion with and without delay and chemotherapy discontinuation stratified by regimen for early breast cancer


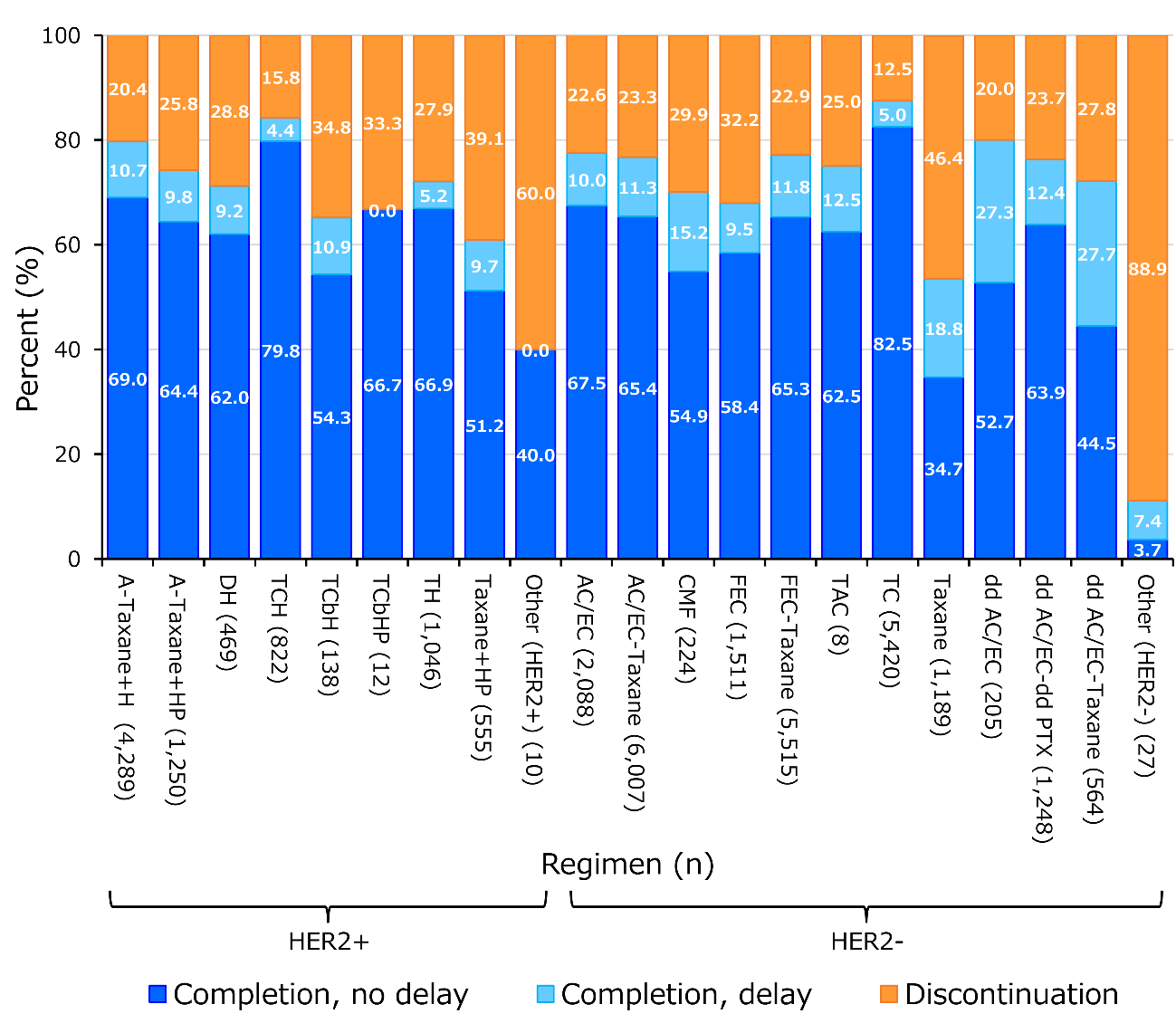


**Abbreviations:** HER2, human epidermal growth factor receptor 2.

**Notes:** The figure shows up to the first two perioperative regimens, and two regimens, if present, were presented with a hyphen (“-“). Regimens were as follows; A: anthracycline; Taxane: docetaxel, docetaxel + cyclophosphamide (TC), paclitaxel (PTX: weekly, every 2 weeks, and every 3 weeks), and nab-PTX (every 3 weeks); H: trastuzumab; HP: trastuzumab + pertuzumab; DH: docetaxel + trastuzumab; TCH: docetaxel + cyclophosphamide + trastuzumab; TCbH: docetaxel + carboplatin + trastuzumab; TCbHP: docetaxel + carboplatin + trastuzumab + pertuzumab; TH: paclitaxel + trastuzumab; AC/EC: doxorubicin/epirubicin + cyclophosphamide; CMF: cyclophosphamide (oral) + methotrexate + fluorouracil; FEC: fluorouracil + epirubicin + cyclophosphamide; TAC: docetaxel + doxorubicin + cyclophosphamide; dd: dose-dense; dd PTX: dose-dense paclitaxel (weekly or every 2 weeks); and dd AC/EC-Taxane: dose-dense doxorubicin/epirubicin + cyclophosphamide - paclitaxel (every 3 weeks or nab-PTX every 3 weeks). The “anthracycline” included AC/EC, dd AC/EC, FEC, and cyclophosphamide (oral) + epirubicin + fluorouracil.

## **Supplementary Figure S2** Timing of chemotherapy for a) ER-positive/HER2-negative, b) ER-positive/HER2-positive, c) ER-negative/HER2-positive, and d) ER-negative/HER2-negative early breast cancer


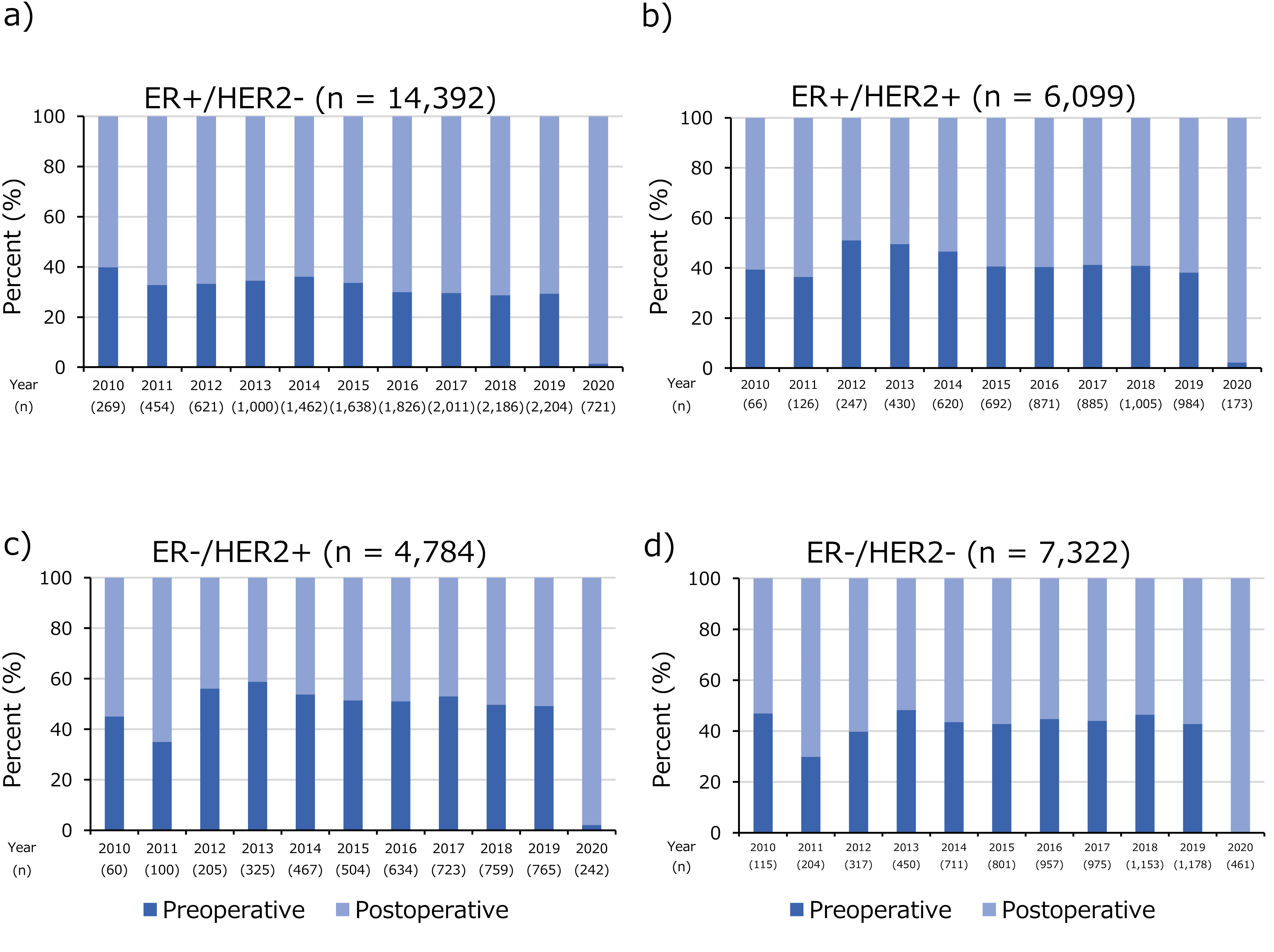


**Abbreviations:** ER, estrogen receptor; and HER2, human epidermal growth factor receptor 2.

## **Supplementary Figure S3** Annual G-CSF use stratified by regimen for a) HER2-positive and b) HER2-negative early breast cancer


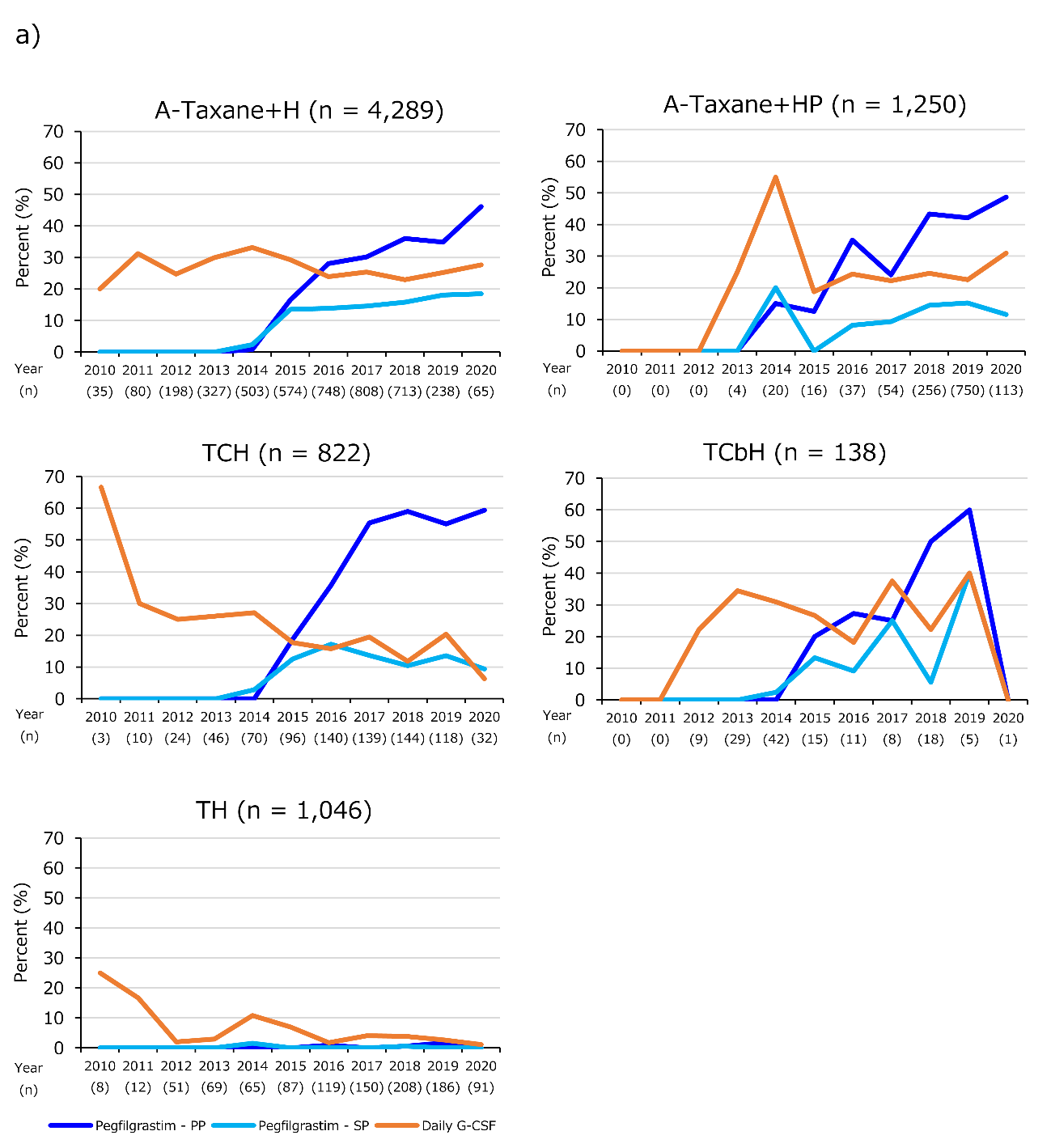


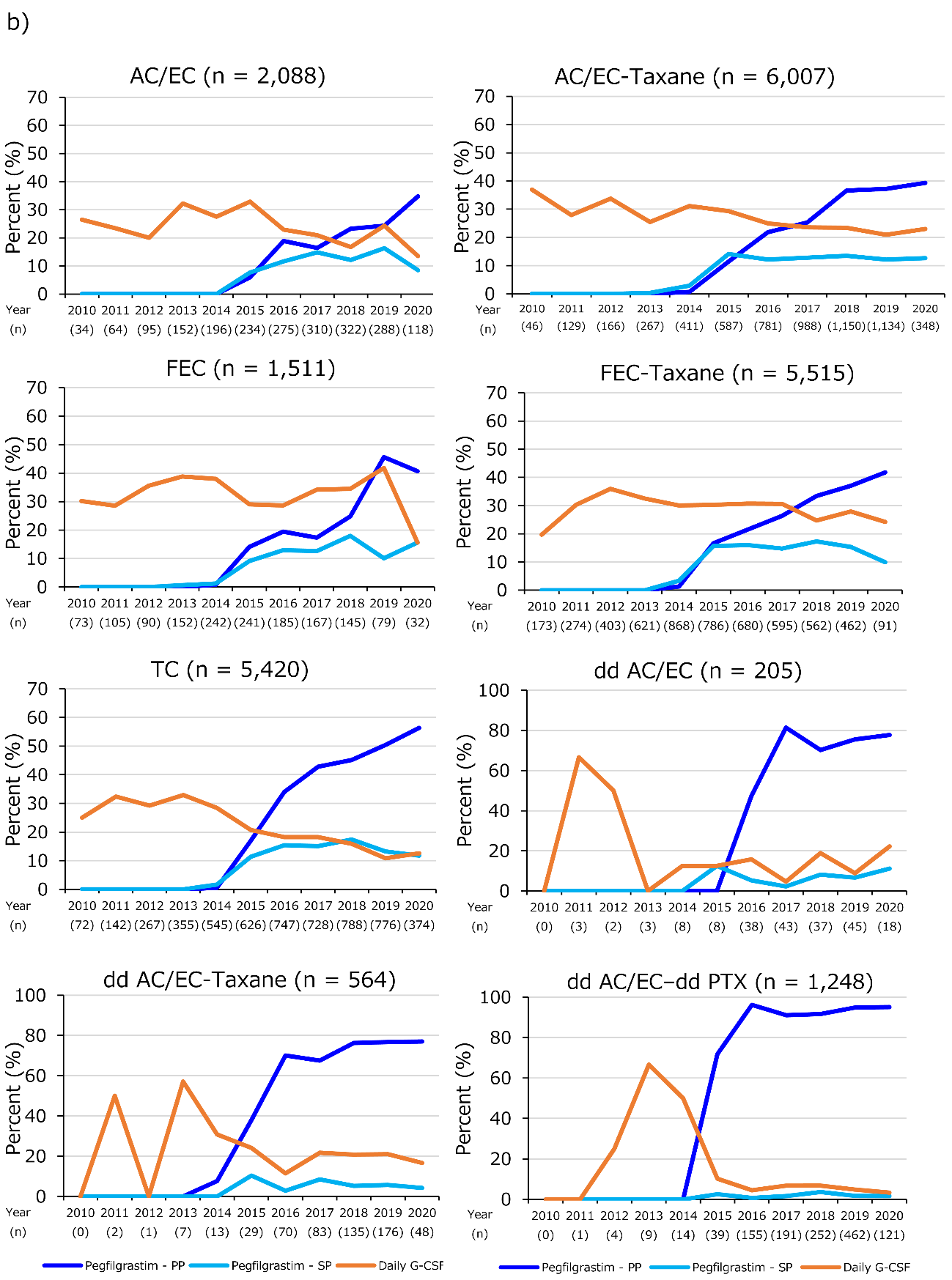


**Abbreviations:** G-CSF, granulocyte colony-stimulating factor; HER2, human epidermal growth factor receptor 2; PP, primary prophylaxis; and SP, secondary prophylaxis.

**Notes:** The figure shows up to the first two perioperative regimens, and two regimens, if present, were presented with a hyphen (“-“). Regimens were as follows; A: anthracycline; Taxane: docetaxel, docetaxel + cyclophosphamide (TC), paclitaxel (PTX: weekly, every 2 weeks, and every 3 weeks), and nab-PTX (every 3 weeks); H: trastuzumab; HP: trastuzumab + pertuzumab; TCH: docetaxel + cyclophosphamide + trastuzumab; TCbH: docetaxel + carboplatin + trastuzumab; TH: paclitaxel + trastuzumab; AC/EC: doxorubicin/epirubicin + cyclophosphamide; FEC: fluorouracil + epirubicin + cyclophosphamide; dd AC/EC-Taxane: dose-dense doxorubicin/epirubicin + cyclophosphamide – paclitaxel (every 3 weeks or nab-PTX every 3 weeks); and dd PTX: dose-dense paclitaxel (weekly or every 2 weeks). The anthracycline included AC/EC, dd AC/EC, FEC, and cyclophosphamide (oral) + epirubicin + fluorouracil.

## **Supplementary Table S2** FN and FNH stratified by regimen for a) HER2-positive and b) HER2-negative early breast cancer

| **a)** | **FN ^a^** | | **FNH** | |
| --- | --- | --- | --- | --- |
| **Regimen** | **n** | **(%)** | **n** | **(%)** |
| **Single regimen** |  |  |  |  |
| TH (n = 818) | 40 | (4.9) | 13 | (1.6) |
| THP (n = 34) | 3 | (8.8) | 0 | (0.0) |
| DH (n = 624) | 153 | (24.5) | 39 | (6.3) |
| DTX+HP (n = 368) | 130 | (35.3) | 37 | (10.1) |
| TCbH (n = 125) | 33 | (26.4) | 13 | (10.4) |
| TCbHP (n= 12) | 0 | (0.0) | 0 | (0.0) |
| TCH (n = 764) | 278 | (36.4) | 61 | (8.0) |
| **Two regimens** |  |  |  |  |
| AC/EC-TH (n = 663) | 230 | (34.7) | 67 | (10.1) |
| AC/EC | 214 | (32.3) | 55 | (8.3) |
| TH | 178 | (26.8) | 16 | (2.4) |
| AC/EC-THP (n = 148) | 33 | (22.3) | 9 | (6.1) |
| AC/EC | 31 | (20.9) | 9 | (6.1) |
| THP | 31 | (20.9) | 1 | (0.7) |
| AC/EC-DH (n = 1,270) | 500 | (39.4) | 98 | (7.7) |
| AC/EC | 403 | (31.7) | 68 | (5.4) |
| DH | 447 | (35.2) | 42 | (3.3) |
| AC/EC-DTX+HP (n = 470) | 156 | (33.2) | 32 | (6.8) |
| AC/EC | 116 | (24.7) | 11 | (2.3) |
| DTX+HP | 146 | (31.1) | 22 | (4.7) |
| dd AC/EC-TH (n = 138) | 71 | (51.4) | 17 | (12.3) |
| dd AC/EC | 70 | (50.7) | 14 | (10.1) |
| TH | 60 | (43.5) | 8 | (5.8) |
| dd AC/EC-THP (n = 47) | 23 | (48.9) | 4 | (8.5) |
| dd AC/EC | 23 | (48.9) | 2 | (4.3) |
| THP | 20 | (42.6) | 3 | (6.4) |
| dd AC/EC-DH (n = 153) | 86 | (56.2) | 13 | (8.5) |
| dd AC/EC | 79 | (51.6) | 7 | (4.6) |
| DH | 79 | (51.6) | 8 | (5.2) |
| dd AC/EC-DTX+HP (n = 126) | 75 | (59.5) | 17 | (13.5) |
| dd AC/EC | 66 | (52.4) | 7 | (5.6) |
| DTX+HP | 70 | (55.6) | 11 | (8.7) |
| FEC-TH (n = 388) | 115 | (29.6) | 32 | (8.2) |
| FEC | 108 | (27.8) | 30 | (7.7) |
| TH | 82 | (21.1) | 4 | (1.0) |
| FEC-THP (n = 18) | 3 | (16.7) | 0 | (0.0) |
| FEC | 3 | (16.7) | 0 | (0.0) |
| THP | 2 | (11.1) | 0 | (0.0) |
| FEC-DH (n = 1,138) | 396 | (34.8) | 186 | (16.3) |
| FEC | 343 | (30.1) | 152 | (13.4) |
| DH | 329 | (28.9) | 90 | (7.9) |
| FEC-DTX+HP (n = 216) | 83 | (38.4) | 35 | (16.2) |
| FEC | 63 | (29.2) | 19 | (8.8) |
| DTX+HP | 71 | (32.9) | 24 | (11.1) |

| **b)** | **FN ^a^** | | **FNH** | |
| --- | --- | --- | --- | --- |
| **Regimen** | **n** | **(%)** | **n** | **(%)** |
| **Single regimen** |  |  |  |  |
| AC/EC (n = 1,999) | 593 | (29.7) | 149 | (7.5) |
| dd AC/EC (n = 202) | 88 | (43.6) | 8 | (4.0) |
| FEC (n = 1,297) | 307 | (23.7) | 111 | (8.6) |
| TC (n = 5,215) | 1,682 | (32.3) | 390 | (7.5) |
| **Two regimens** |  |  |  |  |
| AC/EC-DTX (n = 3,295) | 1,169 | (35.5) | 223 | (6.8) |
| AC/EC | 981 | (29.8) | 167 | (5.1) |
| DTX | 1,009 | (30.6) | 86 | (2.6) |
| AC/EC-nab-PTX (n = 215) | 72 | (33.5) | 12 | (5.6) |
| AC/EC | 68 | (31.6) | 11 | (5.1) |
| nab-PTX | 62 | (28.8) | 1 | (0.5) |
| AC/EC-PTXq1w (n = 1,688) | 531 | (31.5) | 132 | (7.8) |
| AC/EC | 505 | (29.9) | 121 | (7.2) |
| PTXq1w | 374 | (22.2) | 20 | (1.2) |
| dd AC/EC-DTX (n = 392) | 184 | (46.9) | 23 | (5.9) |
| dd AC/EC | 164 | (41.8) | 17 | (4.3) |
| DTX | 161 | (41.1) | 8 | (2.0) |
| dd AC/EC-PTXq1w (n = 577) | 245 | (42.5) | 58 | (10.1) |
| dd AC/EC | 231 | (40.0) | 48 | (8.3) |
| PTXq1w | 212 | (36.7) | 21 | (3.6) |
| dd AC/EC-PTXq2w (n = 548) | 297 | (54.2) | 25 | (4.6) |
| dd AC/EC | 283 | (51.6) | 16 | (2.9) |
| PTXq2w | 289 | (52.7) | 17 | (3.1) |
| DTX-AC/EC (n = 253) | 100 | (39.5) | 16 | (6.3) |
| DTX | 85 | (33.6) | 12 | (4.7) |
| AC/EC | 97 | (38.3) | 9 | (3.6) |
| DTX-FEC (n = 451) | 125 | (27.7) | 6 | (1.3) |
| DTX | 88 | (19.5) | 2 | (0.4) |
| FEC | 117 | (25.9) | 5 | (1.1) |

**Abbreviations:** FN, febrile neutropenia; FNH, febrile neutropenia-related hospitalization; HER2, human epidermal growth factor receptor 2; and ICD-10, International Statistical Classification of Diseases and Related Health Problems, 10th Revision.

**Notes:** The table shows up to the first two perioperative regimens, and two regimens, if present, were presented with a hyphen (“-“). Regimens were as follows; TH: paclitaxel + trastuzumab; THP: paclitaxel + trastuzumab + pertuzumab; DH: docetaxel + trastuzumab; DTX+HP: docetaxel + trastuzumab + pertuzumab; TCbH: docetaxel + carboplatin + trastuzumab; TCbHP: docetaxel + carboplatin + trastuzumab + pertuzumab; TCH: docetaxel + cyclophosphamide + trastuzumab; AC/EC: doxorubicin/epirubicin + cyclophosphamide; dd: dose-dense; FEC: fluorouracil + epirubicin + cyclophosphamide; TC: docetaxel + cyclophosphamide; DTX: docetaxel; nab-PTX: nab-paclitaxel; PTXq1w: paclitaxel weekly; and PTXq2w: paclitaxel every two weeks. A patient with several incidence of FN or FNH was counted once for a given single regimen as well as for two regimens. Where the table showing break-down of two regimens, a patient with incidence of FN or FNH occurring in each of the two regimens was counted in each of the two regimens. Not all combinations are shown in this table.

^a^ FN was defined based on disease codes (ICD-10: D70 and disease code: 8842350).

## **Supplementary Table S3 Baseline characteristics of patients included in the logistic regression model and the OR of FNH**

| **Variables** | **Category** | **Overall** | **FNH** | | **No FNH** | | **OR** | **95% CI** | |
| --- | --- | --- | --- | --- | --- | --- | --- | --- | --- |
|  |  | **n=28,476** | **n=2,102** | | **n=26,374** | |  |  |  |
|  |  | **n** | **n** | **(%)** | **n** | **(%)** |  | **LCL** | **UCL** |
| Regimen ^a^ | AC/EC | 9,748 | 589 | (6.0) | 9,159 | (94.0) | 1.000 | - | - |
|  | FEC | 7,574 | 812 | (10.7) | 6,762 | (89.3) | 1.878 | 1.679 | 2.100 |
|  | DTX+HP | 368 | 37 | (10.1) | 331 | (89.9) | 1.689 | 1.188 | 2.401 |
|  | TCH | 764 | 61 | (8.0) | 703 | (92.0) | 1.399 | 1.060 | 1.846 |
|  | TC | 5,215 | 390 | (7.5) | 4,825 | (92.5) | 1.282 | 1.118 | 1.471 |
|  | DTX | 1,022 | 28 | (2.7) | 994 | (97.3) | 0.430 | 0.292 | 0.632 |
|  | TH | 818 | 13 | (1.6) | 805 | (98.4) | 0.235 | 0.135 | 0.409 |
|  | dd AC/EC | 2,218 | 122 | (5.5) | 2,096 | (94.5) | 0.997 | 0.805 | 1.235 |
|  | TCbH | 125 | 13 | (10.4) | 112 | (89.6) | 1.767 | 0.988 | 3.159 |
|  | DH | 624 | 37 | (5.9) | 587 | (94.1) | 0.959 | 0.680 | 1.351 |
| Age, years | <65 | 21,357 | 1,512 | (7.1) | 19,845 | (92.9) | 1.000 | - | - |
|  | ≥65 | 7,119 | 590 | (8.3) | 6,529 | (91.7) | 1.267 | 1.146 | 1.402 |
| Time from breast cancer surgery to the start of chemotherapy, day | Before surgery | 9,817 | 758 | (7.7) | 9,059 | (92.3) | 0.962 | 0.863 | 1.071 |
|  | ≤30 | 3,157 | 191 | (6.1) | 2,966 | (93.9) | 0.773 | 0.657 | 0.910 |
|  | 31–60 | 11,527 | 877 | (7.6) | 10,650 | (92.4) | 1.000 | - | - |
|  | ≥61 | 3,975 | 276 | (6.9) | 3,699 | (93.1) | 0.931 | 0.801 | 1.081 |
| Comorbidity ^b^ within 1 year before breast cancer diagnosis | 0 | 28,157 | 2,088 | (7.4) | 26,069 | (92.6) | 1.000 | - | - |
|  | 1 | 280 | 13 | (4.6) | 267 | (95.4) | 0.617 | 0.352 | 1.081 |
|  | ≥2 | 39 | 1 | (2.6) | 38 | (97.4) | 0.296 | 0.040 | 2.164 |
| Pegfilgrastim PP | No administration | 22,201 | 1,692 | (7.6) | 20,509 | (92.4) | 1.000 | - | - |
|  | Administration | 6,275 | 410 | (6.5) | 5,865 | (93.5) | 0.878 | 0.777 | 0.992 |
| Postoperative radiation therapy ^c^ | Not performed | 27,790 | 2,060 | (7.4) | 25,730 | (92.6) | 1.000 | - | - |
|  | Performed | 686 | 42 | (6.1) | 644 | (93.9) | 0.824 | 0.587 | 1.156 |

**Abbreviations:** OR, odds ratio; FNH, febrile neutropenia-related hospitalization; CI, confidence interval; LCL, lower confidence limit; UCL, upper confidence limit; and PP, primary prophylaxis.

**Notes:**

^a^ The first chemotherapy regimen was examined in each patient if two regimens were identified, and regimens were as follows: AC/EC: doxorubicin/epirubicin + cyclophosphamide; FEC: fluorouracil + epirubicin + cyclophosphamide; DTX+HP: docetaxel + trastuzumab + pertuzumab; TCH: docetaxel + cyclophosphamide + trastuzumab; TC: docetaxel + cyclophosphamide; DTX: docetaxel; TH: paclitaxel + trastuzumab; dd: dose-dense; TCbH: docetaxel + carboplatin + trastuzumab; and DH: docetaxel + trastuzumab.

^b^ The comorbidity included cardiovascular disease, renal disease, liver disease, diabetes, and human immunodeficiency virus or acquired immunodeficiency syndrome.

^c^ Postoperative radiation therapy was defined as radiation therapy performed ≥5 times from the date of surgery to the date before the initiation of the first chemotherapy or radiotherapy performed ≥5 times during the first chemotherapy after surgery.
